# Impact of hemodialysis on cardiovascular system assessed by pulse wave analysis

**DOI:** 10.1371/journal.pone.0206446

**Published:** 2018-11-02

**Authors:** Malgorzata Debowska, Jan Poleszczuk, Wojciech Dabrowski, Alicja Wojcik-Zaluska, Wojciech Zaluska, Jacek Waniewski

**Affiliations:** 1 Department for Mathematical Modeling of Physiological Processes, Nalecz Institute of Biocybernetics and Biomedical Engineering, Polish Academy of Sciences, Warsaw, Poland; 2 Department of Anesthesiology and Intensive Therapy, Medical University of Lublin, Lublin, Poland; 3 Department of Physical Therapy and Rehabilitation, Medical University of Lublin, Lublin, Poland; 4 Department of Nephrology, Medical University of Lublin, Lublin, Poland; Medizinische Universitat Graz, AUSTRIA

## Abstract

Valuable information about cardiovascular system can be derived from the shape of aortic pulse wave being the result of reciprocal interaction between heart and vasculature. Pressure profiles in ascending aorta were obtained from peripheral waveforms recorded non-invasively (SphygmoCor, AtCor Medical, Australia) before, during and after hemodialysis sessions performed after 3-day and 2-day interdialytic intervals in 35 anuric, prevalent hemodialysis patients. Fluid status was assessed by Body Composition Monitor (Fresenius Medical Care, Bad Homburg, Germany) and online hematocrit monitoring device (CritLine, HemaMetrics, Utah). Systolic pressure and ejection duration decreased during dialysis. Augmentation index remained stable at 30 ± 13% throughout hemodialysis session despite the decrease of augmented pressure and pulse height. Subendocardial viability ratio (SEVR) determined after 3-day and 2-day interdialytic intervals increased during the sessions by 43.8 ± 26.6% and 26.1 ± 25.4%, respectively. Hemodialysis performed after 3-day and 2-day interdialytic periods reduced significantly overhydration by 2.4 ± 1.0 L and 1.8 ± 1.2 L and blood volume by 16.3 ± 9.7% and 13.7 ± 8.9%, respectively. Intradialytic increase of SEVR correlated with ultrafiltration rate (R = 0.39, p-value < 0.01), reduction in overhydration (R = -0.57, p-value < 0.001) and blood volume drop (R = -0.38, p-value < 0.01). The strong correlation between the decrease of overhydration during hemodialysis and increase in SEVR confirmed that careful fluid management is crucial for proper cardiac function. Hemodialysis affected cardiovascular system with the parameters derived from pulse-wave-analysis (systolic and augmented pressures, pulse height, ejection duration, SEVR) being significantly different at the end of dialysis from those before the session. Combination of pulse-wave-analysis with the monitoring of overhydration provides a new insight into the impact of hemodialysis on cardiovascular system.

## Introduction

The relationship between chronic kidney disease (CKD) and cardiovascular disease is bidirectional [1,2]. Cardiovascular disease (including peripheral vascular disease, coronary artery disease or myocardial ischemia) is often present in CKD [1,3]. Inversely—the kidney failure contributes to the cardiovascular disease via deterioration of body fluid management, endothelial dysfunction and vascular calcification; CKD may be a cause and a consequence of arterial hypertension [1,4–6]. Cardiovascular mortality risk in patients receiving dialysis is higher than in general population and the highest among other comorbidities making the efforts to increase our understanding of CKD and hemodialysis treatment effects on cardiovascular system of high importance [2,3,7].

**Fig 1:**
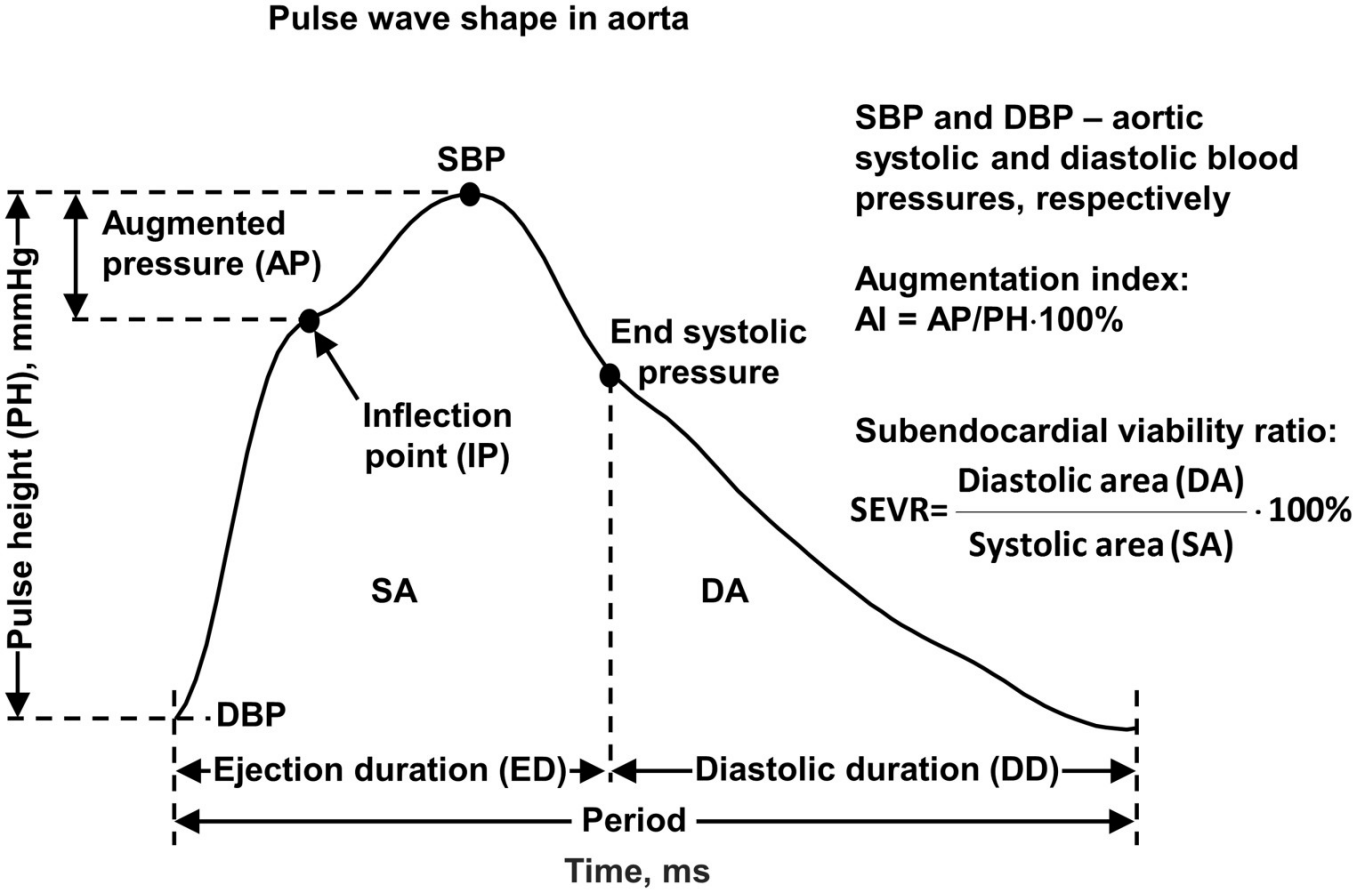
Aortic pulse wave. Most important characteristic landmarks of pulse wave profile with the definitions of parameters derived from the waveform.

With every heartbeat the left ventricle generates pulse (pressure) wave that travels through the arterial tree. Multiple bifurcation points, variable vessel diameter, presence of a plaque, and varying wall elasticity affect the arterial pressure waveform. [8,9]. The shape of pressure wave observed in aorta depends on ventricular-vascular interaction and contains information about the cardiovascular condition [10]. Aortic pressure waveform can be nowadays reconstructed from the peripheral pressure recording using the pulse-wave-analysis (PWA) technique [8,11–13]. The PWA technique is non-invasive, reproducible and provides several parameters that are useful in the assessment of cardiovascular condition [14–16], Fig 1. Systolic and diastolic aortic blood pressures were found to be better indicators of a cardiovascular disease than brachial pressure [9,15,17,18], because aortic pressures represent the true load exerted on vital organs as heart, brain and kidneys [8,19]. The increased effect of arterial waves reflection is a risk factor for all-cause and cardiovascular mortality among hemodialysis patients and in general population [20,21]. Based on aortic pressure wave one can estimate the sufficiency of myocardial blood flow via subendocardial viability ratio (SEVR) [22–24]. The critically low level of the oxygen supply-to-demand ratio, assessed by SEVR, was linked with hypoperfusion and ischemia [24]. The reduction in SEVR was associated with significant increase of cardiovascular mortality in patients with CKD [25]. PWA has been recognized by the international medical societies as a reliable technique in the assessment of cardiovascular status with carotid-femoral pulse wave velocity considered to be the ‘gold-standard’ measurement of arterial stiffness [8,26–29].

Cardiovascular system of a standard hemodialysis patient is under the constant influence of many non-physiological factors. During 2–3 days of interdialytic period patient gains 2–3 L of water that is quickly removed during 4-hour hemodialysis typically performed 3 times per week. Blood flow in extracorporeal circuit during hemodialysis and non-physiologic connection of the vessels of arteriovenous fistula also affect the cardiovascular system. Blood volume and blood pressure decrease during hemodialysis. Hemodialysis is a lifesaving treatment, but at the same time it exerts a considerable load on the cardiovascular system.

The purpose of this study was to assess the impact of different phases of hemodialysis on cardiovascular system by the analysis of pulse wave recorded before, during and after hemodialysis. Parameters derived from the aortic pressure waveform were related to the changes in overhydration and blood volume during hemodialysis.

## Materials and methods

### Ethics statement

The study has been conducted according to the principles expressed in the Declaration of Helsinki, was approved by the Bioethical Committee at the Medical University of Lublin (Poland) and written informed consent was obtained from each patient.

### Patients

Two standard bicarbonate hemodialysis sessions (duration 240.2 ± 13.4 min) were monitored in 35 anuric, prevalent hemodialysis patients (age 61.2 ± 14.3 year, 43% males, dialysis vintage 9.1 ± 8.9 years, body mass index 25.4 ± 5.6 kg/m^2^, Table 1). Patients were selected from a larger cohort of 60 subjects according to the eligibility for PWA measurements. Exclusion criteria included: accelerated or mechanically controlled irregular heart rhythms, arrhythmias, atrial fibrillation or flutter, significant aortic valve stenosis and unstable carotid plaques that might rupture upon massage. 49% of 35 selected patients did not take any antihypertensive medications and 26% of patients took more than 2 antihypertensive drugs. Five selected patients had symptoms of congestive heart failure and 8 patients had peripheral artery disease. Seven patients had diabetes. The cause of end stage renal disease was: chronic glomerulonephritis (confirmed by renal biopsy) in 19, obstructive nephropathy in 5, tubulointerstitial nephropathy in 3, diabetes nephropathy in 1 patient, and other/unknown in 7 patients. There were 4 current smokers, 2 former smokers and 83% of patients never smoked cigarettes. All patients underwent their regular treatment, kept taking their medications and did not change their dietary habits. Participants were asked to restrain from coffee, cigarettes, heavy meals and physical exercises for at least 30 minutes before measurements. In all of the patients both monitored hemodialysis sessions were performed at the same time of the day. Each patient used the same type of dialyzer for both monitored dialysis sessions. Membrane material, effective surface area (m^2^), ultrafiltration coefficient (mL/h x mmHg) and sterilization method were: polysulfone based, 1.8, 59, inline steam in 12 patients; polysulfone based, 1.6, 16, inline steam in 6 patients; polyethersulfone composition, 1.7, 18, Gamma-ray in 6 patients; polysulfone based, 2.1, 17, Gamma-ray in 4 patients; polysulfone based, 1.6, 6.4, ethylene oxide in 4 patients; and polyamix (polyarylethersulfone, polyvinylpyrrolidone and polyamide blend), 2.1, 15, steam in 3 patients, respectively. The average flow of blood and dialysis fluid was 287.3 ± 47.4 mL/min (range 180–380 mL/min) and 500 mL/min, respectively. All patients had arteriovenous fistulas.

**Table 1:** Basic characteristics of the studied group of patients with biochemical measurements in blood serum at the end of midweek hemodialysis session.

	Mean ± SD (N = 35)	Range
Gender, % male	43%	-
Age, year	61.2 ± 14.3	32–85
Height, cm	167.9 ± 9.4	148–185
Weight, kg	72.2 ± 19.9	39.0–139.6
Body mass index, kg/m^2^	25.4 ± 5.6	14.5–44.1
*Biochemical measurements in serum*		
Potassium, mmol/L	4.00 ± 0.35	3.6–5.0
Sodium, mmol/L	139.94 ± 1.95	136–144
Calcium, mg/dL	9.43 ± 0.62	8.2–10.9
Inorganic phosphate, mg/dL	2.47 ± 0.73	1.3–4.6
Urea, mg/dL	39.02 ± 13.02	20.3–74.1
Urea KT/V	1.18 ± 0.18	0.83–1.62

The study protocol did not allow patients to eat during hemodialysis. During intradialytic time the following medications were given: nonsteroidal anti-inflammatory drug in 5, iron sucrose in 2, and darbepoetin alfa and low molecular weight heparin in 1 of 70 monitored sessions. In one case 200 mL of NaCl 0.9% was given and the patient was disconnected from the dialyzer 10 min before the prescribed time because of cramps. No hypotension events requiring medical intervention were observed.

### Pulse wave analysis

**Fig 2:**
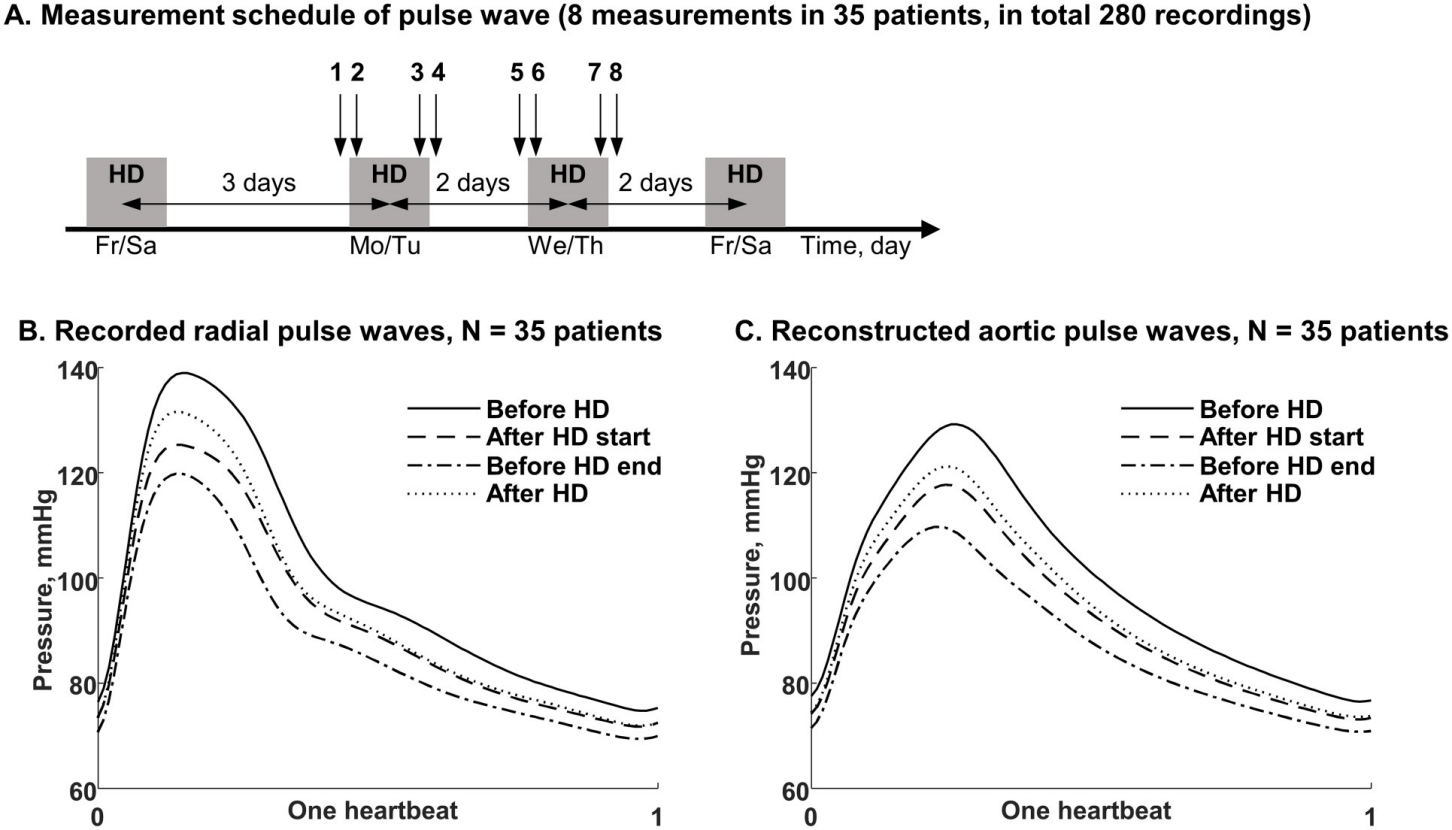
Measurement schedule and average peripheral and aortic pulse wave profiles. (A) Pressure profile was recorded 8 times in each of 35 patients before the start, after the start, before the end and after the end of hemodialysis (HD) performed after 3-day and 2-day interdialytic periods. Presented are (B) average recorded peripheral and (C) reconstructed aortic pulse waves scaled to the one heartbeat with pooled data for hemodialysis performed after 3-day and 2-day interdialytic intervals. Characteristic points of pulse waveform shown in Fig 1 are less noticeable here due to averaging of individual profiles. For the parameter values see S1 Table.

Pulse wave shape in radial artery was recorded using applanation tonometry (SphygmoCor, AtCor Medical, Australia) before the start, after the start, before the end, and after the end of two hemodialysis sessions performed after 3- and 2-day interdialytic intervals in patients in restful state, Fig 2A and 2B. In 28 patients all 8 PWA measurements were performed within one week and in 7 patients two monitored hemodialysis sessions were in two different weeks with the longest break of 2 weeks in-between. All measurements were made in at least duplicate and the recording with highest quality (defined and calculated by SphygmoCor software as ‘*operator index’*) was chosen. Measurements with insufficient quality (‘*operator index’* ≤ 74) were excluded according to the producer’s indication. All recordings were performed by one trained clinician in the non-fistula arm. The radial pulse wave was calibrated to the blood pressure measured oscillometrically at brachial artery (Omron M3, Omron Healthcare, Kyoto, Japan). The aortic pulse pressure waveform was derived from the recorded peripheral waveform using the generalized transfer function through the built-in device software, Fig 2B and 2C.

Augmentation index was determined as augmented pressure (AP) over pressure height (PH), AI = AP/PH·100% with PH being the difference between aortic systolic (SBP) and diastolic (DBP) pressures (PH = SBP–DBP), Fig 1. There are two definitions of AI: AI = AP/PH·100% or AI2 = PH/(PH-AP)·100%. In device specific reports and also in the literature both values (AI and AI2) can be found what leads to misunderstandings, e.g., AI = 30% corresponds to the AI2 = 143%, as AI2 = 1/(1-AI). Throughout this study we use AI.

Ejection duration (ED) is the time from the start of the pulse to the closure of the aortic valve that determines the end of systole. Ejection duration together with diastolic time (DD) constitute the period, which is the inverse of heart rate (HR), Fig 1.

SEVR–subendocardial viability ratio–was defined as diastolic time index (DTI) over tension time index (TTI): SEVR = DTI/TTI with DTI = meanDBP·DD·HR and TTI = meanSBP·ED·HR, where meanDBP and meanSBP are average aortic pressures during diastole and systole, respectively. Geometrically, SEVR can be determined as the diastolic area over systolic area of aortic pulse pressure, Fig 1.

### Monitoring of body composition

Fluid status was assessed by whole-body bioimpedance (Body Composition Monitor, Fresenius Medical Care, Bad Homburg, Germany). Overhydration, extracellular, intracellular and total body water (being the sum of extra- and intra-cellular volumes) were measured before and after each hemodialysis session [30].

Relative changes of blood volume were measured by online monitor (CritLine, HemaMetrics, Utah) during both hemodialysis sessions after 3-day and 2-day interdialytic periods. The volume of blood (BV) at the end of dialysis was calculated using an anthropometric formula (BV = 28.5·height + 31.6·weight—2820 for males and BV = 16.52 ·height + 38.46·weight—1369 for females, height in cm, weight in kg, BV in mL) [31]. Pre-dialytic blood volume was recalculated from its final value using the drop of blood volume measured by CritLine.

### Statistical analysis

The data are presented as mean ± standard deviation (SD) and statistical significance was set at the level of p-value < 0.05, unless otherwise indicated. Statistical dependence between variables was tested using Spearman’s correlation coefficient (R). Multiple comparisons were investigated by Wittkowski test followed by multiple pairwise comparison analysis based on adjusted Scheffe’s procedure. Wittkowski test is a Friedman-type statistics for consistent multiple comparisons for unbalanced designs with missing data [32]. In our dataset we have 25 missing records (among 280) in pulse wave measurements, 3 (among 140) in data of body composition and 3 (among 70) for blood volume. Changes in parameters were considered significant if statistical significance was present in at least one of the hemodialysis sessions. Statistical analysis was performed in MATLAB R2017b equipped with Statistics and Machine Learning Toolbox (MathWorks, Natick, MA, USA).

## Results

### Changes of pulse wave shape during hemodialysis

**Fig 3:**
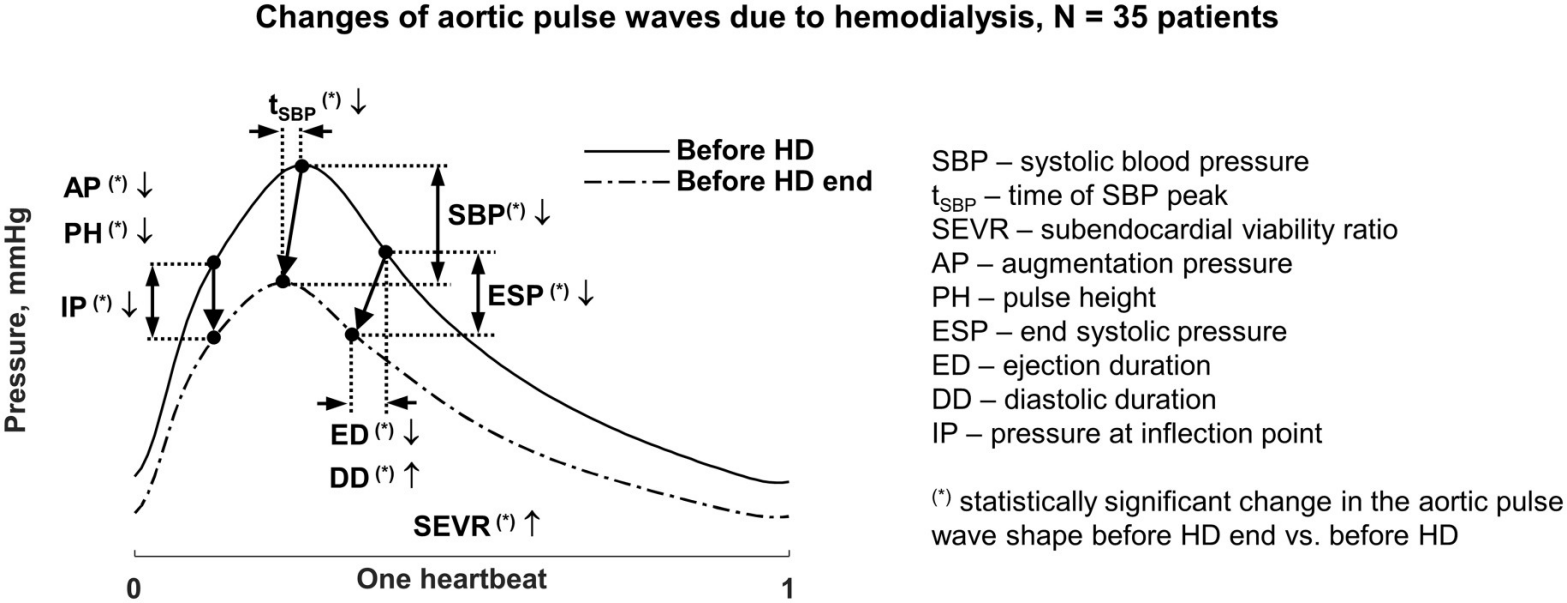
Statistically significant changes in the aortic pulse wave shape caused by hemodialysis. Changes of aortic pulse wave when comparing profile before the start and right before the end of hemodialysis session. The time was scaled to one heartbeat and pooled data for hemodialysis performed after 3-day and 2-day interdialytic are presented. For the parameter values see S1 Table.

Hemodialysis did not affect the heart rate which had a stable average value of 69 ± 12 beats/min. No hemodialysis-related changes were detected in brachial and aortic diastolic blood pressures with averages 73.7 ± 13.2 mmHg and 74.5 ± 13.2 mmHg, respectively, Fig 2B and 2C. The systolic pressure (brachial and aortic, SBP) dropped after the start of dialysis and was decreasing until the end of dialysis with a significant reduction of about 20 ± 22 mmHg from before the start to before the end of hemodialysis session, Figs 2B and 2C and 3. This drop was accompanied by the decrease in the time to the systolic peak from the wave foot (t_SBP_) of 9.7%, aortic end systolic pressure (ESP) of 11%, pressure at the inflection point (IP) of 11.8%, and estimated ejection duration (ED) of 13.6%, Fig 3. Ending hemodialysis session and unplugging the dialyzer, however, caused the rebound of all of those values (SBP, tSBP, ESP, IP, ED) towards the state observed after the start of dialysis, compare Figs 2B and 2C and 3. See S1 Table for the detailed values of the parameters derived from the pulse wave profiles.

### Hemodialysis impact on cardiovascular biomarkers

**Fig 4:**
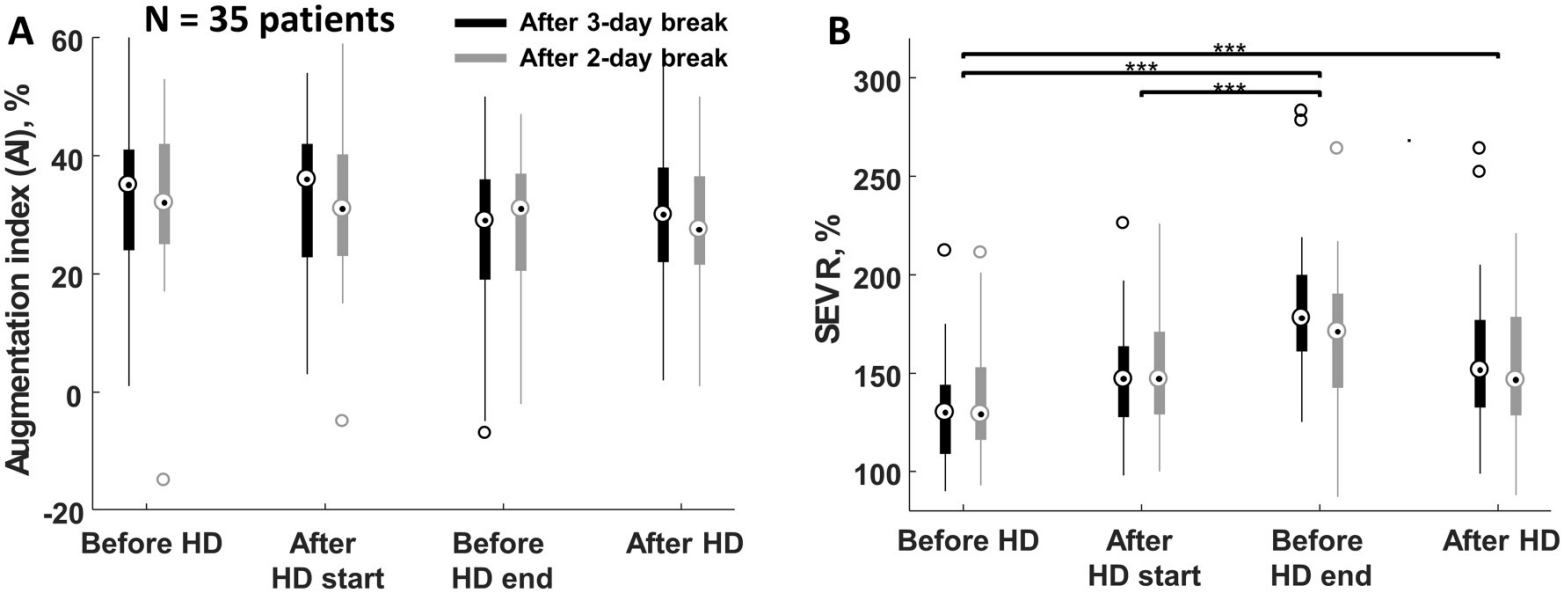
Augmentation index and subendocardial viability ratio before, during and after hemodialysis. (A) Augmentation index and (B) subendocardial viability ratio (SEVR) before the start, after the start, before the end and after the end of hemodialysis session (HD) performed after 3-day and 2-day interdialytic intervals. Statistically significant difference with p-value < 0.001 was marked as ‘***’.

The augmented pressure (AP) and pulse height (PH) decreased during hemodialysis by 34.4 ± 53.2% and 26.4 ± 24.5%, respectively (Figs 2C and 3), but the augmentation index (AI = AP/PH) did not change due to the treatment and remained stable around 30 ± 13%, Fig 4A, S1 Table. AI correlated positively with patient age (R = 0.41, p-value < 0.05) and negatively with patient height (R = -0.62, p-value < 0.001).

SEVR determined after 3-day and 2-day interdialytic intervals increased during the session by 43.8 ± 26.6% and 26.1 ± 25.4%, respectively (comparison of states before the start and before the end of hemodialysis) and dropped after hemodialysis by 13.9 ± 10.1% and 10.1 ± 12.3%; nevertheless, it was significantly higher than before the start of dialysis, Fig 4B.

### Hemodialysis impact on body fluids

The set ultrafiltration volumes were 2.96 ± 0.73 L and 2.35 ± 0.97 L for hemodialysis sessions carried out after 3-day and 2-day interdialytic intervals (p-value < 0.001), what corresponded to ultrafiltration rates of 12.33 ± 2.94 mL/min and 9.73 ± 3.85 mL/min, respectively. Hemodialysis performed after 3-day and 2-day interdialytic periods reduced overhydration by 2.4 ± 1.0 L and 1.8 ± 1.2 L (p-value < 0.001), respectively, as assessed by body composition monitor; and blood volume decreased by 16.3 ± 9.7% and 13.7 ± 8.9% (p-value < 0.001), respectively, Table 2. Extracellular water volume decreased and intracellular water remained at steady level during hemodialysis, Table 2.

**Table 2:** Weight, blood volume and water pools of the body after 3-day and 2-day interdialytic periods.

	After 3-day interdialytic break		After 2-day interdialytic break		Global
	Before HD	After HD	Before HD	After HD	p-value^(^^a^^)^
Weight, kg	75.1 ± 20.0	72.4 ± 19.9***	74.3 ± 20.4^#^	72.2 ± 19.9***	<0.001
Blood volume (BV), L	5.1 ± 1.2	4.2 ± 0.9***	4.9 ± 1.1	4.2 ± 0.9***	<0.001
Overhydration (OH), L	2.9 ± 1.8	0.5 ± 1.7***	2.4 ± 2.3	0.6 ± 2.4***	<0.001
Extracellular water, L	18.2 ± 3.6	16.1 ± 3.6***	17.7 ± 4.0	15.9 ± 3.7***	<0.001
Intracellular water, L	17.5 ± 4.0	18.5 ± 5.0	17.7 ± 4.3	17.9 ± 4.4	<0.001
Total body water, L	35.7 ± 7.2	34.7 ± 8.2**	35.4 ± 7.8	33.9 ± 7.5**	<0.001

^a^ Global p-value is provided for all 4 measurement points.

p—value:

*** < 0.001,

** < 0.01 vs. before HD,

^#^ < 0.05 vs. 3-day interdialytic interval

### Changes in SEVR correlate with overhydration shifts

**Fig 5:**
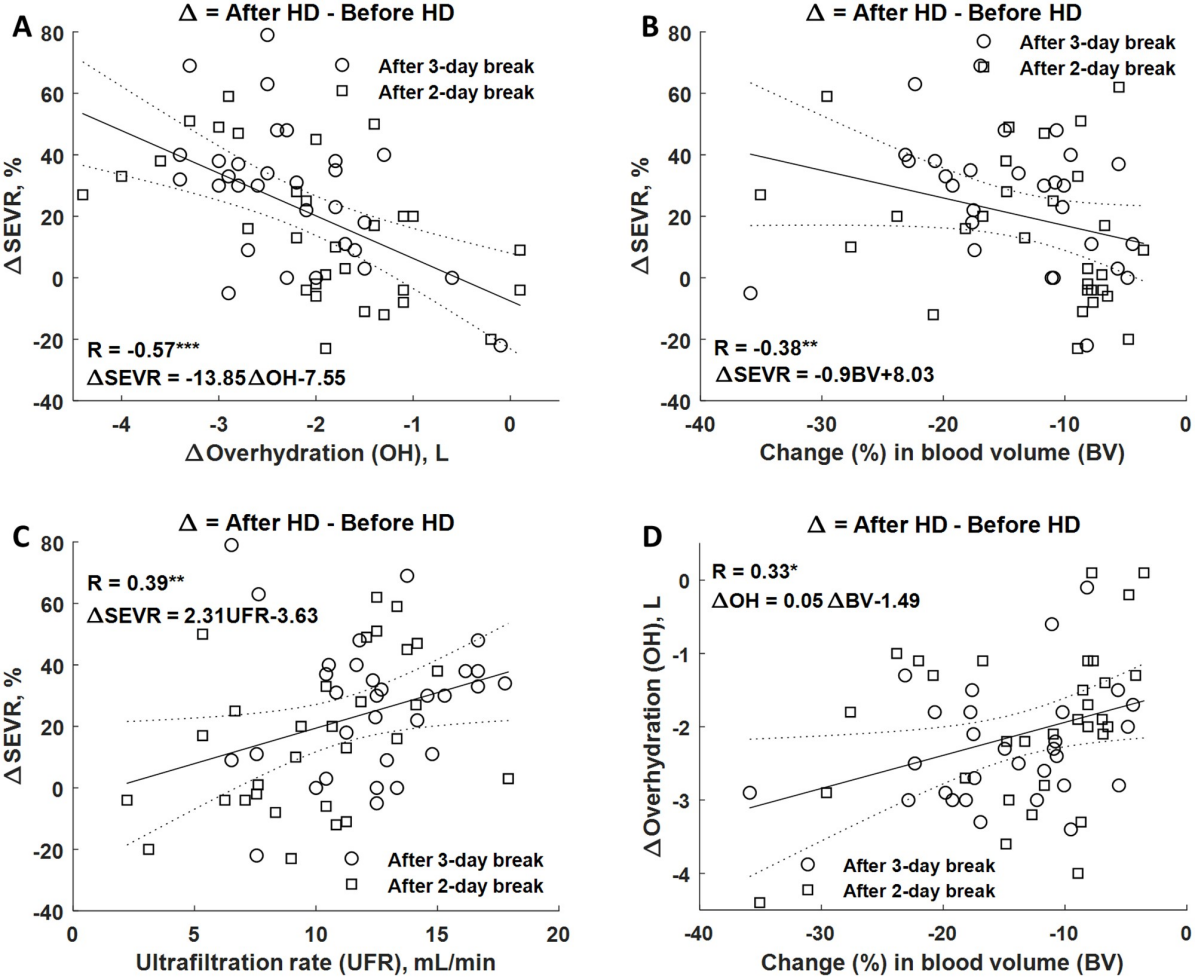
Correlation between absolute changes in SEVR and changes of overhydration, reduction of blood volume and ultrafiltration rate. Correlation between the change in subendocardial viability ratio (SEVR) and the change of overhydration (OH), (A), the percentage change in blood volume (BV), (B), and ultrafiltration rate (C). Shown is also correlation between overhydration (OH) and the percentage change in blood volume (BV) during hemodialysis (HD), (D). Symbols ‘***’, ‘**’, and ‘*’ denote p-value < 0.001, p-value < 0.01, and p-value < 0.05, respectively.

Changes of SEVR correlated negatively with changes in overhydration (R = -0.57, p-value < 0.001) when considering difference between values after vs. before hemodialysis, Fig 5A. Increase of SEVR during hemodialysis correlated also with the drop of blood volume, Fig 5B (R = -0.38, p-value < 0.01). Intradialytic change of SEVR was associated with ultrafiltration rate, Fig 5C (R = 0.39, p-value < 0.01). Drop of blood volume correlated positively with the reduction in overhydration, Fig 5D (R = 0.33, p-value < 0.05).

## Discussion

The pulse wave analysis (PWA) technique is a non-invasive and useful tool to investigate the cardiovascular state. The accuracy of the PWA estimated aortic blood pressure was validated but also questioned by some studies [8,33–37]. Our approach, however, partially overcomes this issue as we assess the impact of hemodialysis on cardiovascular system considering the relative changes of pulse wave parameters during the treatment using multiple longitudinal measurements.

We observed the most pronounced changes in PWA-derived parameters when comparing measurements performed before the start and before the end of hemodialysis session. This behavior is clearly visible in SEVR value, which was the smallest before hemodialysis with the highest value before hemodialysis termination, Fig 4B. The increase of SEVR due to hemodialysis was on average 43.1 ± 31.8% and 23.4 ± 26.2% when comparing pretreatment values with those obtained shortly before the end and after the end of hemodialysis session, respectively (pooled data for hemodialysis performed after 3-day and 2-day interdialytic intervals), compare Fig 4B. Aortic systolic pressure (SBP) and end systolic pressures (ESP) had the highest values before hemodialysis, which decreased during the session achieving the smallest values before the end of hemodialysis, Figs 2C and 3. After the end of hemodialysis the cardiovascular system had a tendency to return towards the pre-dialysis state, but remained typically at the level reached just after hemodialysis start, Figs 2B and 2C and 4B. Our study clearly showed that for some parameters (e.g. SEVR, SBP, ED) the timing of pulse wave measurement is important with the time just before the end of hemodialysis seems to be the critical with parameters of cardiovascular system being much different from those before the session, Fig 3.

We did not observe the statistically significant differences between the patient parameters measured after 3-day vs. 2-day breaks (except for weight), although the change in numerical values were in agreement with intuitive expectation, Table 2. Parameters of pulse wave after 3-day vs. 2-day interdialytic periods were not statistically different either, S1 Table. This lack of difference was partly due to the conservative statistical test for multiple comparisons, partly to the low difference in overhydration after 3-day and 2-day breaks, and partly to the high interpatient variability. The intradialytic SEVR change correlated stronger with the change of overhydration than with ultrafiltration rate. During hemodialysis overhydration decreases and this change is expected to correspond with the ultrafiltration volume and subsequently with the ultrafiltration rate (if treatment time is fixed). However, overhydration (the excess of body fluid) estimated by body composition monitor in relation to a reference group may not exactly reflect the volume, which is set to be removed during dialysis. The opposite sign of correlation coefficient R between ΔSEVR and ΔOH vs. ΔSEVR and ultrafiltration rate (compare Fig 5A and 5C) is because ultrafiltration is expressed as positive value in agreement with its standard description. The average value of augmentation index AI, which describes the reflective properties of the arterial tree, of about 30 ± 13% was similar to that obtained by other researchers in patients with end stage renal disease [38–41], Fig 4A. According to our analysis AP and PH decreased during hemodialysis but AI, being their ratio, was not affected by the treatment, Figs 3 and 4A. Previous studies on AI mostly showed its reduction during dialysis and a gradual increase during interdialytic interval [38–41], although the study by Covic et al. [41] has shown the increase of AI in one subgroup when comparing pre- vs. post-dialytic values. In our study we observed the increase of AI in 39% and the decrease in 61% of patients but on average the intradialytic change of AI was not statistically significant, Fig 4A.

Hemodialysis affected myocardial perfusion assessed by SEVR–subendocardial viability ratio–that is considered an estimate of subendocardial oxygen supply related to oxygen demand [22,24,42]. Previous studies on SEVR reported increase of SEVR during hemodialysis session and its gradual reduction during interdialytic interval [38,43]; this observation is confirmed in our study, but in addition we show the correlation between the magnitude of SEVR increase and the drop of overhydration (R = -0.57, p-value < 0.001), Fig 5A. Similar relationship was found for the changes in SEVR and in blood volume, Fig 5B. The correlation of SEVR with changes in blood volume was weaker than with changes in overhydration possibly due to the plasma refilling during hemodialysis, i.e., a mechanism that counteracts the drop in blood volume through the inflow of fluid into the vascular bed [44]. The increase of SEVR found in our study suggests the improvement of oxygen supply-to-demand ratio during hemodialysis.

Two recent studies showed, however, a decrease in myocardial perfusion during hemodialysis [45,46]. Myocardial perfusion dropped during hemodialysis in all 7 patients by around 27% on average when studied by PET [46], and in 7 out of 12 patients studied with magnetic resonance imaging [45]. The subendocardial blood flow is typically much reduced or stopped during systole, so the perfusion of subendocardial muscle is restricted mostly to diastole [24]. The main driving force for myocardial perfusion during diastole is the pressure in the ascending aorta. According to our data, the average aortic blood pressure during diastole is on average stable during hemodialysis session with some tendency to decrease, and actually it was found decreasing in around 70% but increasing in 30% of dialysis sessions, Table 3. Thus, the drop in coronary blood flow and myocardial perfusion may be expected in part of the patients, as observed in direct measurements [45,46]. Wave-intensity wall analysis and tissue velocity imaging demonstrated some improvement in the systolic function, while diastolic variables were found to be more load dependent [47]. Myocardial stunning is frequent during hemodialysis [48]. Using echocardiographic and tissue Doppler imaging it was shown that hemodialysis deteriorates cardiac diastolic function indices and improves pulmonary circulation load, but systolic function is not changed [49]. A recent review noticed that the results of echocardiographic studies on the acute effect of hemodialysis are not consistent, but most of them show that cardiac chamber size and pulmonary circulation loading decrease during dialysis (pre- vs. post-hemodialysis), diastolic function is worsen but systolic function does not change [50].

**Table 3:** Parameters of aortic pulse wave (mean ± SD) derived before start, after start, before end and after end of hemodialysis (HD) performed after 3-day and 2-day interdialytic intervals, compare Figs 1 and 2.

**After 3-day interdialytic break**	**Before HD**	**After HD start**	**Before HD end**	**After HD**	
*Measurement points*:	**1**	**2**	**3**	**4**	
Mean diastolic pressure (meanDBP), mmHg	89.7 ± 14.8	85.8 ± 14.6	81.0 ± 16.7	86.4 ± 14.1	
Mean systolic pressure (meanSBP), mmHg	115.0 ± 18.9^(3)^	106.5 ± 21.3	97.9 ± 20.0^(1)^	107.2 ± 17.1	
meanDBP/meanSBP	0.783 ± 0.061^(3)^	0.812 ± 0.071	0.829 ± 0.054^(1)^	0.810 ± 0.080	
Diastolic duration (DD), ms	551.4 ± 128.2^(3)^	596.6 ± 147.2	634.0 ± 150.0^(1)^	588.9 ± 140.3	
Ejection duration (ED), ms	331.6 ± 31.9^(3,4)^	326.7 ± 33.3^(3,4)^	284.2 ± 41.9^(1,2)^	298.8 ± 36.8^(1,2)^	
DD/ED	1.66 ± 0.33^(3,4)^	1.82 ± 0.37^(3)^	2.23 ± 0.43^(1,2,4)^	1.97 ± 0.43^(1,3)^	
Heart rate (HR), beats/min	69.8 ± 11.6	67.1 ± 12.1	67.9 ± 14.2	69.8 ± 12.7	
Diastolic time index (DTI), mmHg	56.0 ± 9.3	55.2 ± 10.1	56.0 ± 11.9	57.2 ± 9.4	
Tension time index (TTI), mmHg	44.4 ± 9.4^(3,4)^	38.9 ± 8.9^(3)^	31.2 ± 7.7^(1,2,4)^	37.3 ± 8.7^(1,3)^	
Subendocardial viability ratio (SEVR), %	129.9 ± 26.8^(3,4)^	145.9 ± 29.2^(3)^	183.8 ± 36.7^(1,2)^	158.4 ± 36.2^(1)^	
**After 2-day interdialytic break**	**Before HD**	**After HD start**	**Before HD end**	**After HD**	**Global**
*Measurement points*:	**5**	**6**	**7**	**8**	**p-value**
Mean diastolic pressure (meanDBP), mmHg	88.4 ± 11.9	82.4 ± 12.7	80.7 ± 16.4	84.5 ± 15.1	0.001
Mean systolic pressure (meanSBP), mmHg	112.0 ± 16.4^(6,7)^	101.8 ± 16.9^(5)^	97.9 ± 19.5^(5)^	106.1 ± 19.0	<0.001
meanDBP/meanSBP	0.794 ± 0.069^(7)^	0.814 ± 0.068	0.830 ± 0.096^(5)^	0.802 ± 0.085	<0.001
Diastolic duration (DD), ms	549.9 ± 123.3	601.3 ± 122.2^(8)^	578.4 ± 128.9	557.9 ± 116.1^(6)^	<0.001
Ejection duration (ED), ms	322.7 ± 33.5^(7)^	321.2 ± 37.0^(7)^	284.8 ± 42.5^(5,6)^	296.1 ± 41.9	<0.001
DD/ED	1.71 ± 0.36^(7)^	1.88 ± 0.35	2.030 ± 0.36^(5)^	1.89 ± 0.35	<0.001
Heart rate (HR), beats/min	70.4 ± 10.9	66.5 ± 10.0^(7,8)^	71.9 ± 14.5^(6)^	72.3 ± 13.3^(6)^	<0.001
Diastolic time index (DTI), mmHg	55.7 ± 7.9	53.7 ± 7.9	54.1 ± 10.6	55.4 ± 10.6	0.556
Tension time index (TTI), mmHg	42.5 ± 8.4^(6,7)^	36.4 ± 8.0^(5)^	33.3 ± 8.5^(5)^	37.9 ± 8.7	<0.001
Subendocardial viability ratio (SEVR), %	135.3 ± 30.1^(7)^	151.6 ± 28.8	167.9 ± 35.7^(5)^	151.2 ± 34.8	<0.001

The measurement points statistically different (with p-value < 0.05) from the current data are shown in superscript brackets. Global p-value is for all 8 measurement points.

Why SEVR increases considerably during dialysis? SEVR is the ratio of diastolic time index, DTI, and tension time index, TTI, see Methods. DTI may remain stable during dialysis even if diastolic pressure decreases because the diastolic duration increases, Table 3. In contrast, the pressure decrease in systole during hemodialysis is accompanied by the decrease in systolic duration and TTI decreases, Table 3. It is important to notice that the decrease of myocardial perfusion is concomitant with the extension of diastolic time, and therefore the total blood (and oxygen) supply to the myocardium per heartbeat may not necessarily fall during dialysis. Therefore, SEVR increases during dialysis mostly because the total workload on the left ventricular muscle decreases, not necessarily because the myocardial perfusion increases. Thus, we may say that the oxygen supply-to-demand ratio for the left ventricular muscle may improve during the hemodialysis session, even if the myocardial perfusion would decrease. All these considerations deal with the “average” behavior but, as noted by Buchanan et al. [45], much interpatient variability exists. Another interpretation of the increase in SEVR is provided if one applies a different form of the equation for SEVR: SEVR = (meanDBP/meanSBP)·(DD/ED), c.f. Methods for the definitions of DTI and TTI. Both factors in this formula, (meanDBP/meanSBP) and (DD/ED), increase during dialysis if considered separately, Table 3. This means that there are two favorable factors for the increase in SEVR: 1) mean systolic pressure decreases faster (on average) that mean diastolic pressure (the workload decreases more than possible reduction in myocardial perfusion), and 2) diastolic duration (i.e. the time for heart muscle rest and perfusion) increases and ejection duration (the time for heart muscle work) decreases, Table 3. Further studies are needed regarding the applicability of SEVR in the assessment of myocardial perfusion, compare [24].

In conclusion, the profile of pressure wave in aorta is the result of ventricular-arterial interaction and is a reliable source of information about cardiovascular system. This study is the first to provide the comprehensive analysis of parameters derived from pulse-wave-analysis and their changes caused by hemodialysis treatment. We show the significant decrease of systolic (SBP), end systolic (ESP), augmented pressures (AP) and pulse height (PH) during hemodialysis. Time of the systolic pressure peak (t_SBP_) and ejection duration (ED) decreased, whereas diastolic duration (DD) increased and period (1/HR) remained unchanged. Augmentation index (AI) did not change during the session. Intradialytic increase in SEVR–subendocardial viability ratio–correlated with ultrafiltration rate, the reduction in overhydration (OH) and blood volume (BV). During hemodialysis session we traced and discussed components of SEVR showing significant decrease of the tension time index (TTI—the area under the curve of aortic pressure during systole) and stable value of diastolic time index (DTI–the area of aortic pressure during diastole). The estimation of SEVR from the aortic waveform is of importance for clinical monitoring of patients as an unfavorable imbalance between oxygen supply and demand may reduce heart perfusion below a critical value. A limitation of our study is the lack of comparison between SEVR and its components with an alternative method able to assess myocardium during hemodialysis. Several essential parameters derived from pulse wave registered shortly before the end of hemodialysis were considerably different from those before the session, however, after the end of hemodialysis the cardiovascular system had tendency to return towards the pre-dialysis state. Pulse wave analysis combined with the monitoring of body fluid have the potential to be a diagnostic tool to assess the impact of hemodialysis on the cardiovascular system.

## Supporting information

S1 Table Parameters of pulse wave derived before, during and after hemodialysis performed after 3-day and 2-day interdialytic intervals. (DOCX)
