# Supplementary material for: Impact of hemodialysis on cardiovascular system assessed by pulse wave analysis
Source: PLoS One. 2018 Nov 2;13(11):e0206446. doi: 10.1371/journal.pone.0206446 (PMC6279117; doi:10.1371/journal.pone.0206446)
Supplement: S1 Table — (DOCX) [file pone.0206446.s001.docx]

S1 Table. Parameters of pulse wave derived before, during and after hemodialysis performed after 3-day and 2-day interdialytic intervals. Parameters of pulse wave (mean ± SD) derived before the start, after the start, before the end and after the end of hemodialysis (HD) performed after 3-day and 2-day interdialytic intervals, compare Figs 1-2. In superscript brackets shown are the measurement points statistically different (with p-value < 0.05) from the current data. Global p-value is provided for all 8 measurement points.

| **After 3-day interdialytic break** | **Before HD** | **After HD start** | **Before HD end** | **After HD** |  |
| --- | --- | --- | --- | --- | --- |
| *Measurement point:* | **1** | **2** | **3** | **4** |  |
| Brachial systolic pressure, mmHg | 143.8 ± 24.9^(3)^ | 131.3 ± 27.3 | 122.5 ± 25.2^(1)^ | 134.1 ± 22.2 |  |
| Brachial diastolic pressure, mmHg | 76.8 ± 13.3 | 74.2 ± 13.5 | 70.5 ± 13.9 | 74.5 ± 12.9 |  |
| Mean pressure, mmHg | 100.3 ± 16.2^(3)^ | 94.1 ± 16.5 | 87.3 ± 17.8^(1)^ | 94.4 ± 14.9 |  |
| Aortic systolic pressure (SBP), mmHg | 132.4 ± 23.1^(3)^ | 122.2 ± 26.6 | 110.8 ± 24.1^(1)^ | 122.8 ± 20.8 |  |
| Aortic diastolic pressure (DBP), mmHg | 77.9 ± 13.4 | 75.0 ± 13.5 | 71.1 ± 14.3 | 75.5 ± 13.1 |  |
| Aortic end systolic pressure (ESP), mmHg | 116.3 ± 19.4 | 109.3 ± 20.3 | 102.1 ± 22.4 | 110.8 ± 17.1 |  |
| Aortic augmented pressure (AP), mmHg | 18.2 ± 9.7^(3)^ | 16.5 ± 11.1 | 11.6 ± 9.8^(1)^ | 14.9 ± 10.2 |  |
| Aortic pulse height (PH), mmHg | 54.5 ± 17.6^(3)^ | 47.2 ± 20.6 | 39.6 ± 15.3^(1,4)^ | 47.3 ± 17.5^(3)^ |  |
| Aortic augmentation index (AI), % | 32.9 ± 13.2 | 32.9 ± 12.7 | 26.2 ± 15.6 | 30.0 ± 12.5 |  |
| Time of aortic peak blood pressure (t_SBP_), ms | 230.6 ± 30.0^(3,4)^ | 231.9 ± 25.6^(3)^ | 199.6 ± 39.7^(1,2)^ | 214.3 ± 25.1^(1)^ |  |
| Period, ms | 883.1 ± 148.1 | 923.4 ± 168.8 | 918.4 ± 179.1 | 887.8 ± 161.2 |  |
| Ejection duration/Period, % | 38.2 ± 4.5^(3,4)^ | 36.1 ± 4.7^(3)^ | 31.5 ± 4.1^(1,2,4)^ | 34.3 ± 4.6^(1,3)^ |  |
| Diastolic duration/Period, % | 61.8 ± 4.5^(3,4)^ | 63.9 ± 4.7^(3)^ | 68.5 ± 4.1^(1,2,4)^ | 65.7 ± 4.6^(1,3)^ |  |
| Pressure at inflection point, mmHg | 114.4 ± 19.1^(3)^ | 105.8 ± 19.3 | 98.9 ± 18.7^(1)^ | 107.9 ± 17.4 |  |
| Time of inflection point, ms | 107.4 ± 19.3 | 105.5 ± 14.0 | 106.8 ± 22.7 | 102.4 ± 15.3 |  |
| **After 2-day interdialytic break** | **Before HD** | **After HD start** | **Before HD end** | **After HD** | **Global** |
| *Measurement point:* | **5** | **6** | **7** | **8** | **p-value** |
| Brachial systolic pressure, mmHg | 140.1 ± 22.1^(6,7)^ | 125.8 ± 22.5^(5)^ | 121.7 ± 25.5^(5)^ | 135.6 ± 27.2 | <0.001 |
| Brachial diastolic pressure, mmHg | 75.8 ± 11.3 | 72.4 ± 11.2 | 70.7 ± 14.9 | 72.1 ± 13.3 | 0.257 |
| Mean pressure, mmHg | 98.3 ± 13.2^(6,7)^ | 90.2 ± 14.0^(5)^ | 87.4 ± 16.9^(5)^ | 93.2 ± 15.9 | <0.001 |
| Aortic systolic pressure (SBP), mmHg | 128.9 ± 20.4^(6,7)^ | 116.0 ± 20.3^(5)^ | 110.8 ± 23.9^(5)^ | 122.2 ± 24.1 | <0.001 |
| Aortic diastolic pressure (DBP), mmHg | 77.1 ± 11.3 | 73.1 ± 11.3 | 71.5 ± 15.1 | 73.4 ± 13.4 | 0.057 |
| Aortic end systolic pressure (ESP), mmHg | 113.8 ± 16.0^(7)^ | 104.0 ± 16.1 | 101.2 ± 19.6^(5)^ | 109.3 ± 19.3 | <0.001 |
| Aortic augmented pressure (AP), mmHg | 17.4 ± 10.4 | 13.2 ± 6.9 | 11.7 ± 10.0 | 14.1 ± 9.4 | <0.001 |
| Aortic pulse height (PH), mmHg | 51.9 ± 17.3^(7)^ | 42.8 ± 14.4 | 39.2 ± 19.7^(5)^ | 48.7 ± 20.8 | <0.001 |
| Aortic augmentation index (AI), % | 32.7 ± 13.9 | 31.1 ± 12.6 | 27.7 ± 12.7 | 27.8 ± 11.7 | 0.057 |
| Time of aortic peak blood pressure (t_SBP_), ms | 225.3 ± 33.6^(7)^ | 224.7 ± 33.8^(7)^ | 202.5 ± 33.3^(5,6)^ | 210.3 ± 33.0 | <0.001 |
| Period, ms | 872.6 ± 140.5 | 922.7 ± 142.4^(7,8)^ | 863.2 ± 159.4^(6)^ | 854.1 ± 142.2^(6)^ | <0.001 |
| Ejection duration/Period, % | 37.6 ± 5.0^(7)^ | 35.2 ± 4.4 | 33.4 ± 3.9^(5)^ | 35.1 ± 4.3 | <0.001 |
| Diastolic duration/Period, % | 62.4 ± 5.0^(7)^ | 64.8 ± 4.3 | 66.6 ± 3.9^(5)^ | 64.9 ± 4.3 | <0.001 |
| Pressure at inflection point, mmHg | 111.0 ± 15.9^(7)^ | 103.3 ± 17.2 | 99.1 ± 18.9^(5)^ | 108.0 ± 19.7 | <0.001 |
| Time of inflection point, ms | 106.6 ± 17.2 | 117.0 ± 39.4^(8)^ | 101.7 ± 14.8 | 103.5 ± 18.8^(6)^ | <0.001 |
